# Supplementary material for: Size-Exclusion Chromatography as a Technique for the Investigation of Novel Extracellular Vesicles in Cancer
Source: Cancers (Basel). 2020 Oct 27;12(11):3156. doi: 10.3390/cancers12113156 (PMC7693800; doi:10.3390/cancers12113156)
Supplement: Supplementary file 1 [file cancers-12-03156-s001.pdf]

Size Exclusion Chromatography as a Technique for the Investigation of Novel Extracellular Vesicles in Cancer

Daniel S. K. Liu, Flora M. Upton, Eleanor Rees, Christopher Limb, Long R. Jiao, Jonathan Krell and Adam E. Frampton

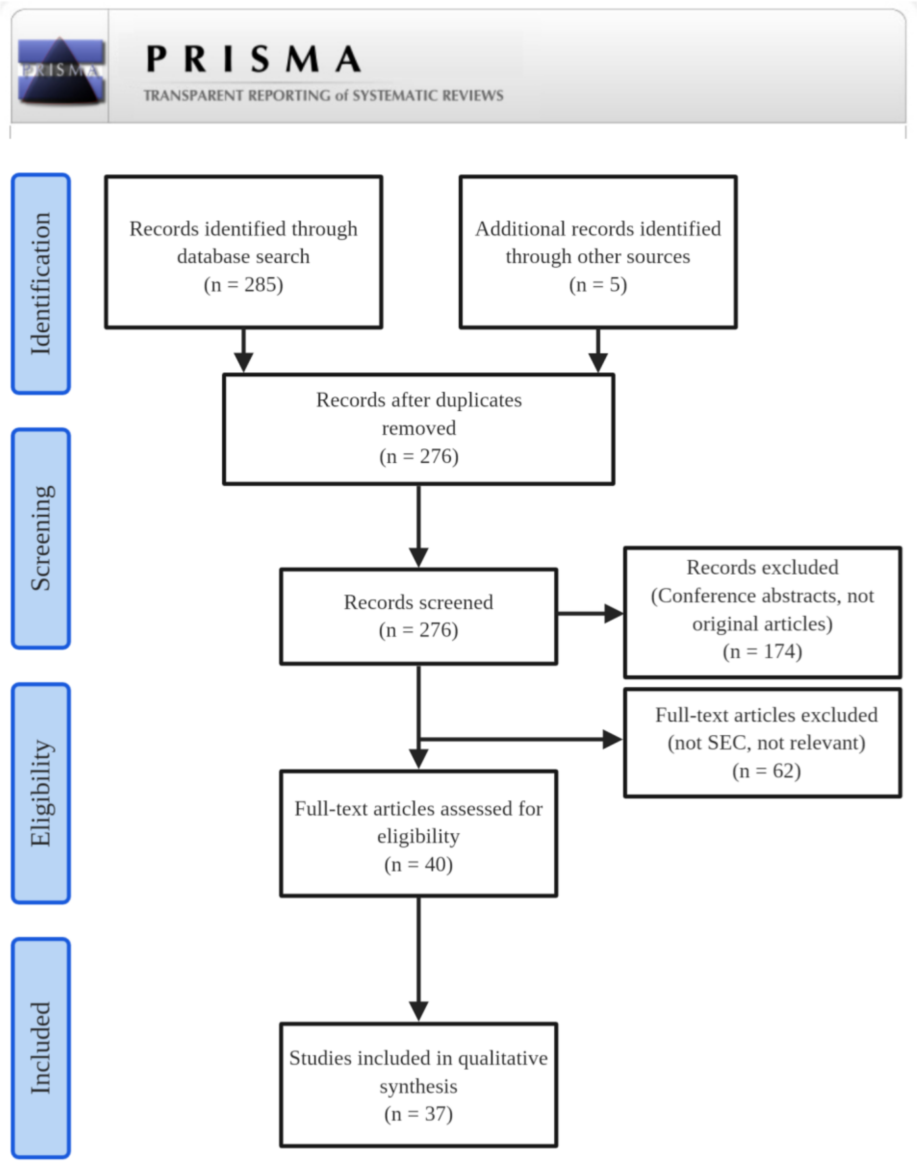

Figure S1. PRISMA Flow Chart.

Table S1. Size-exclusion methodology compared in 37 papers reviewed (ordered chronologically).

Authors & Reference	Publication Title	Pub. Year	Biofluids	Preparation Method	Loading Volume	Method of Isolation	Concentration Step
Taylor et al. [67]	T-cell apoptosis and suppression of T-cell receptor/CD3-zeta by Fas ligand-containing membrane vesicles shed from ovarian tumours	2003	Human serum	Sample clotted and centrifuged (400× *g* for 10 min)	500 μL	Bio-Gel A50m column (1.5 × 45 cm)	UC (100,000× *g* for 1 h)
Altanerova et al. [2]	Fas ligand-positive membranous vesicles isolated from sera of patients with oral cancer induce apoptosis of activated T lymphocytes.	2005	Human serum; CCM	Not described	500 μL	Sepharose 2B in 1.0 × 35 cm column	UC (105,000× *g* for 1 h)
Wieckowski et al. [78]	Tumour-derived microvesicles promote regulatory T cell expansion and induce apoptosis in tumour-reactive activated CD8+ T lymphocytes.	2009	Human serum; CCM	Not described	-	Sepharose 2B in 1.0 × 35 cm column	UC (105,000× *g* for 1 h)
Rabinowits et al. [65]	Exosomal microRNA: a diagnostic marker for lung cancer.	2009	Human plasma	Not described	1 mL	Sepharose 2B	Magnetic immunoaffinity to EpCAM
Davies et al. [93]	Microfluidic filtration system to isolate extracellular vesicles from blood.	2012	Mouse whole blood	Not described	Up to 240 μL	Microfluidic platform involving photopatterned porous polymer monoliths	*n*/A
Muller et al. [32]	Isolation of biologically-active exosomes from human plasma.	2014	Human plasma	Differential centrifugation (1,000× *g* for 10 min, 10,000× *g* for 30 min) followed by filtration (0.22 μm)	9 mL	Sepharose 2B in an A50m column	UC (105,000× *g* for 2 h)
Hong et al. [40]	Plasma exosomes as markers of therapeutic response in patients with acute myeloid leukaemia.	2014	Human plasma	Differential centrifugation (1,000× *g* for 10 min), filtration (0.22 μm) and repeat centrifugation (10,000× *g* for 30 min)	9 mL	Sepharose 2B in an A50m column	UC (100,000× *g* for 2 h)
Schuler et al. [80]	Human CD4+ CD39+ regulatory T cells produce adenosine upon co-expression of surface CD73 or contact with CD73+ exosomes or CD73+ cells.	2014	Human plasma	Differential centrifugation (1,000× *g* for 10 min, 10,000× *g* for 10 min) followed by filtration (0.22 μm)	5 mL	Sepharose 2B in an A50m column	UC (100,000× *g* for 3 h)
Lobb et al. [41]	Optimized exosome isolation protocol for cell culture supernatant and human plasma.	2015	CCM	Clarified CCM was prepared by 300× *g* for 10 min, then filtration (0.22 μm). Subsequent concentration to 500μL with either pressure-driven (Stirred Cell) or centrifugation-based (Centricon Merck Millipore, Burlington, MA, USA) methods.	500μL	qEV column (Izon)	Pooled and concentrated to 200 μL with Amicon Ultra-4 (10 kDa) (Merck Millipore, Burlington, MA, USA)
			Human plasma	Differential centrifugation (1,200× *g* for 10 min, then 1,800× *g* for 10 min) before storage. After thaw differential centrifugation (1,500× *g* for 10 min, then 10,000× *g* for 20 min)	1mL		Filtration (Ultrafree 0.22 μm) (Merck Millipore, Burlington, MA, USA) then concentration to 200 μL with Amicon Ultra-4 (10kDa) (Merck Millipore)
Welton et al. [89]	Ready-made chromatography columns for extracellular vesicle isolation from plasma.	2015	CCM	Differential centrifugation (400× *g* for 10 min, then 2,000× *g* for 15 min) and filtration (0.22 μm)	1mL	Exo-Spin Midi Columns (Cell Guidance Systems, Cambridge, UK)	UC (200,000× *g* for 2 h) or by a precipitation method Exosome precipitant, from the Exo-Spin

			Human plasma	Blood plasma obtained with 400× *g* spin for 7 min, then 6,000× *g* for 10 min and filtration (0.22 μm)	Up to 1.5 mL		kit (Cell Guidance Systems, St. Luis, MO, USA)
Hong et al. [69]	Isolation of biologically active and morphologically intact exosomes from plasma of patients with cancer.	2016	Human plasma	1,000× *g* for 10 min before storage. Differential centrifugation (2,000× *g* for 10 min, then 10,000–14,000× *g* for 30 min) and filtration (0.22 μm)	0.5–1 mL	Mini-SEC (Sepharose 2B in 1.5cm × 12cm mini columns)	VivaSpin 500 (300,000 MWCO; Sartorius, Göttingen, Germany)
Sakha et al. [63]	Exosomal microRNA miR-1246 induces cell motility and invasion through the regulation of DENND2D in oral squamous cell carcinoma.	2016	CCM	Filtration (0.45 μm), then concentration with Amicon Ultra-15 (10 KDa) at 5,000× *g* for 70 min	400 μL	Sephacryl S-400 in a 5 mL column	Not described
van Eijndhoven et al. [33]	Plasma vesicle miRNAs for therapy response monitoring in Hodgkin lymphoma patients.	2016	Human plasma	Differential centrifugation (900× *g* 7 min, then 2,500× *g* for 10 min, then 500× *g* for 10min)	1.5 mL	Sepharose CL/2B in a 10 mL column bed volume	n/A
			Human serum	Differential centrifugation (1,710× *g* for 7 min, then 500× *g* for 10 min)			
Djusberg et al. [43]	High levels of the AR-V7 Splice Variant and Co-Amplification of the Golgi Protein Coding YIPF6 in AR Amplified Prostate Cancer Bone Metastases.	2017	CCM; Human plasma	Centrifugation (3,000× *g* for 30 min) then filtration (0.45 μm), and concentration with Amicon-15 (100 kDa) filter	500 μL	qEV column (Izon)	n/A
Kawakami et al. [31]	Gamma-glutamyltransferase activity in exosomes as a potential marker for prostate cancer.	2017	Human serum	Centrifugation at 1800× *g* before freezing	500 μL	EVSecond (GL Science, Tokyo, Japan)	n/A
Ludwig et al. [81]	Suppression of Lymphocyte Functions by Plasma Exosomes Correlates with Disease Activity in Patients with Head and Neck Cancer.	2017	Human plasma	1,000× *g* for 10 min before storage. Differential centrifugation (2,000× *g* for 10 min, then 14,000× *g* for 30 min) and filtration (0.22 μm)	1mL	Mini-SEC (Sepharose 2B in 1.5 × 12cm mini columns)	VivaSpin 500 (300,000 MWCO)
Suarez et al. [87]	A bead-assisted flow cytometry method for the semi-quantitative analysis of Extracellular Vesicles.	2017	CCM	Differential centrifugation (400× *g* for 5 min and 2,000× *g* for 10 min) and concentration with Amicon-15 filter	1.5mL	Sepharose CL-2B in a 20 mL syringe	n/A
Guerreiro et al. [1]	Efficient extracellular vesicle isolation by combining cell media modifications, ultrafiltration, and size-exclusion chromatography.	2018	CCM	Differential centrifugation (4,000× *g* for 5 min, then 15,000× *g* for 45 min) then concentration using Amicon-Ultra 15 (30 kDa, 50 kDa, and 100 kDa)	4mL	Sepharose CL-2B in 30mL column	Immunoaffinity capture of CD9+ used for flow cytometry
Ludwig et al. [82]	Exosomes from HNSCC Promote Angiogenesis through Reprogramming of Endothelial Cells.	2018	CCM	Differential centrifugation (2,000× *g* for 10 min, then 10,000× *g* for 10 min) followed by filtration (0.22 μm), and concentration with Vivacell 100 (100,000 MWCO)	1mL	Mini-SEC (Sepharose 2B in 1.5 × 12cm mini columns)	Amicon Ultra 0.5 mL (100kDa)
			Human plasma	Differential centrifugation (1,000× *g* for 10 min before storage, after thaw 2,000× *g* for 10 min, then 10,000× *g* for 30 min) and filtration (0.22 μm)			

Peacock et al. [64]	Extracellular vesicle microRNA cargo is correlated with HPV status in oropharyngeal carcinoma.	2018	CCM	Differential centrifugation (300× *g* for 10 min, 2000× *g* for 10 min, 10,000× *g* for 30 min) then concentrated to 0.5 mL using Vivaspin-20 (100 kDa MWCO)	500 µL	Sepharose CL-2B in Econo-Pac columns (Bio-rad, Hercules, California, USA)	UC (100,000× *g* for 1 h)
Smith et al. [92]	Integrated nanoscale deterministic lateral displacement arrays for separation of extracellular vesicles from clinically-relevant volumes of biological samples.	2018	Human serum and urine	Diluted 1:4, labelled with SYBRgold and filtered (0.22 µm)	500 µL	Microfluidic platform involving a nanoscale deterministic lateral displacement (nanoDLD) array	n/A
			Human serum and urine	Centrifugation (14,000 RPM for 20 min)	500 µL	qEVoriginal (Izon)	Concentration with Corning (50K MWCO)
Theodoraki et al. [46]	Separation of plasma-derived exosomes into CD3((+)) and CD3((-)) fractions allows for association of immune cell and tumour cell markers with disease activity in HNSCC patients.	2018	Human plasma	Differential centrifugation (1,000× *g* for 10 min before storage, after thaw 2,000× *g* for 10 min, then 10,000× *g* for 30 min) and filtration (0.22 µm)	1mL	Mini-SEC (Sepharose 2B in 1.5cm x 12cm mini columns)	Vivaspin 500 (300,000 MWCO) or captured with streptavidin magnetic beads
Theodoraki et al. [83]	Plasma-derived Exosomes Reverse Epithelial-to-Mesenchymal Transition after Photodynamic Therapy of Patients with Head and Neck Cancer.	2018	Human plasma	Differential centrifugation (1,000× *g* for 10 min before storage, after thaw 2,000× *g* for 10 min, then 14,000× *g* for 30 min) and filtration (0.22 µm)	1 mL	Mini-SEC (Sepharose 2B in 1.5 × 12cm mini columns)	Vivaspin 500 (300,000 MWCO) or captured with streptavidin magnetic beads
Theodoraki et al. [47]	Clinical Significance of PD-L1(+) Exosomes in Plasma of Head and Neck Cancer Patients.	2018	Human plasma	Differential centrifugation (1,000× *g* for 10 min before storage, after thaw 2,000× *g* for 10 min, then 10,000× *g* for 30 min) and filtration (0.22 µm)	1 mL	Mini-SEC (Sepharose 2B in 1.5cm x 12cm mini columns)	Vivaspin 500 (300,000 MWCO) or captured with streptavidin magnetic beads
Abramowicz et al. [53]	Ionizing radiation affects the composition of the proteome of extracellular vesicles released by head-and-neck cancer cells in vitro.	2019	CCM	Differential centrifugation (200× *g* for 10min, 2,000× *g* for 10 min, 10,000× *g* for 30min), filtration (0.22 µm), and concentration with Vivacell 100 (Sartorius, Göttingen, Germany	1 mL	qEVoriginal (Izon)	Vivaspin 500 (10,000 MWCO)
Altanerova et al. [2]	Prodrug suicide gene therapy for cancer targeted intracellular by mesenchymal stem cell exosomes.	2019	CCM	Centrifugation (800× *g* for 5 min) and filtration (0.22 µm)	Not described	Sepharose CL-2B column or Sephacryl 500 High Resolution column	n/A
Broggi et al. [44]	Tumor-associated factors are enriched in lymphatic exudate compared to plasma in metastatic melanoma patients.	2019	CCM	Differential centrifugation (300× *g* for 10min, 2000× *g* for 10min, 10,000× *g* for 20min) then concentration with Amicon Ultra-15	500 µL	qEVoriginal (Izon)	n/A
Czystowska-Kuzmicz et al. [11]	Small extracellular vesicles containing arginase-1 suppress T-cell responses and promote tumor growth in ovarian carcinoma.	2019	Human plasma; Human ascites	Differential centrifugation (500× *g* for 10 min, 2,500× *g* for 20min) and filtration (0.22 µm)	500 µL	qEV column(Izon)	Millipore (100,000 MWCO) or captured with magnetic beads to CD9, CD63, or CD81
Dong et al. [92]	Efficient isolation and sensitive quantification of extracellular vesicles based on an integrated ExoID-Chip using photonic crystals.	2019	Human serum; CCM	Centrifugation (3000× *g* for 5 min) and diluted 1:9	200 µL	Integrated microfluidic chip (ExoID-Chip)	n/A
Freitas et al. [55]	Different isolation approaches lead to diverse glycosylated extracellular vesicle populations.	2019	CCM	Centrifugation (800× *g* for 5 min, 2,000× *g* for 10 min), filtration (0.22	500 µL	qEV (IZON)	Amicon Ultra-15 (10kDa MWCO)

				μm) then UC (100 000× *g* for 16 h, then 100,000× *g* for 2 h wash)			
Indira Chandran et al. [59]	Ultrasensitive Immunoprofiling of Plasma Extracellular Vesicles Identifies Syndecan-1 as a Potential Tool for Minimally Invasive Diagnosis of Glioma.	2019	Human plasma	Centrifugation (2,000× *g* for 10 min) prior to storage	500 μL	qEV columns (IZon)	n/A
Lane et al. [23]	Optimizing Size Exclusion Chromatography for Extracellular Vesicle Enrichment and Proteomic Analysis from Clinically Relevant Samples.	2019	CCM EVs spiked into human plasma	CCM (800× *g* for 5 min, then 10 000× *g* for 30 min), then concentrated using Amicon Ultra-15 (50 kDa MWCO)<br>Plasma (800× *g* for 5 min, 12 000× *g* for 45 min)	200–500 μL	Sepharose CL-2B and Sepharose 4B in 10 mL columns	Not described
Lennon et al. [3]	Single molecule characterization of individual extracellular vesicles from pancreatic cancer.	2019	CCM	CCM (300× *g* for 10 min) concentrated using Vivaspin 20 (100 kDa MWCO)	400μL	qEV original (IZon)	n/A
			Human plasma	*n/A*	~200 μL		
Ludwig et al. [4]	Optimization of cell culture conditions for exosome isolation using mini-size exclusion chromatography (mini-SEC).	2019	CCM	Differential centrifugation (2,000× *g* for 10 min, then 10,000× *g* for 30 min), filtration (0.22 μm) and concentration using Vivacell 100	1 mL	Mini-SEC (Sepharose 2B in 1.5 × 12cm mini columns)	0.5 mL Amicon Ultra (100 kDa)
Ludwig et al. [57]	Proteomes of exosomes from HPV(+) or HPV(-) head and neck cancer cells: differential enrichment in immunoregulatory proteins.	2019	CCM	Differential centrifugation (2,000× *g* for 10 min, then 10,000× *g* for 30 min), filtration (0.22 μm) and concentration using Vivacell 100	1 mL	Mini-SEC (Sepharose 2B in 1.5 × 12cm mini columns)	Vivaspin 500 (100,000 MWCO)
Sjoqvist et al. [86]	Oral keratinocyte-derived exosomes regulate proliferation of fibroblasts and epithelial cells.	2019	CCM	Differential centrifugation (300× *g* for 10 min, 3000× *g* for 10 min) and concentration using Amicon Ultra-14 and Ultra-4 (100 kDa then 10 kDa)	500 μL	qEV Original (Izon)	Amicon Ultra-4 (10 kDa)
Theodoraki et al. [48]	Circulating exosomes measure responses to therapy in head and neck cancer patients treated with cetuximab, ipilimumab, and IMRT.	2019	Human plasma	Differential centrifugation (1,000× *g* for 10 min before storage, after thaw 2,000× *g* for 10 min, then 10,000× *g* for 30 min) and filtration (0.22 μm)	1 mL	Mini-SEC (Sepharose 2B in 1.5 × 12cm mini columns)	Vivaspin 500 (100,000 MWCO)

Abbreviations: min - minutes, h - hour, CCM - Cell Conditioned Media, EpCAM - Epithelial cell adhesion molecule, kDa - kilodaltons, MWCO - Molecular Weight Cut-off, UC – Ultracentrifugation.

Table S2. Papers reviewed showing EV characterization (ordered by focus of study).

Authors & Reference	Year	Cancer Investigated	Source: Cell Line	Source: Sample Size	Characterization Methods: Protein Composition: EV-Enriched	Protein Composition: Non-EV Enriched	Single vesicle Visualization (EM)	Size And Concentration (NTA)	Findings
**Proteomic Studies**									
Taylor et al. [67]	2003	Ovarian Cancer	-	OvCa (*n* = 11), Controls (*n* = 13)	MHC Class I, FasL	No	No	No	Fas ligand-containing membrane vesicles elevated in serum from ovarian cancer patients and reduced expression of the zeta chain in T lymphocytes leading to T-cell apoptosis.
Altanerova et al. [2]	2005	Oral Squamous cell carcinoma	PCI-13	OSCC (*n* = 27), Control (*n* = 20)	FasL	No	Yes	No	FasL positive microvesicles in sera were correlated with T stage, able to induce death in activated T cells but this was also shown in FasL-negative patients.
Hong et al. [40]	2014	Acute Myeloid Leukaemia	-	AML (*n* = 16), Controls (*n* = 7)	CD9, CD81, LAMP-1	No	Yes	No	Changes in exosomal protein and/or TGF-β1 content were altered in response to AML chemotherapy.
Djusberg et al. [43]	2017	Castration-resistant Prostate cancer	22Rv1	CRPC (*n* = 3), Controls (*n* = 3)	No	No	No	NanoSight 300 (Malvern Panalytical, Malvern, UK)	EVs from YIPF6-overexpressing 22Rv1 cells contained tissue factor and other coagulation factors decreasing clotting time in plasma
Kawakami et al. [31]	2017	Prostate Cancer	-	CRPC (*n* = 6), CSPC (*n* = 31), Control BPH (*n* = 8)	CD9, PSMA, β-actin and GGT1	No	No	No	GGT1 exosomal expression correlated with PC but in vivo EVs isolated by UC and not SEC . UC EVs showed no clear association in PC patients
Theodoraki et al. [46]	2018	Head and Neck Squamous cell carcinoma	-	HNSCC (*n* = 22), Controls (*n* = 6)	TSG 101	No	Yes	qNano (Izon Science, Christchurch, New Zealand)	CD3+ exosomes represented a higher proportion (50%) in HNSCC patients compared to 20–30% in healthy donors. High levels of CD44v3 on CD3− exosomes were associated with unfavourable clinicopathological parameters.
Theodoraki, et al. [47]	2018	Head and Neck Squamous cell	-	HNSCC (*n* = 40)	No	No	No	No	Exosomal PD-L1 were associated with disease activity and with clinical stage but not with serum PD-L1 levels. There was also

		carcinoma							dose-dependent inhibition of CD8+ effector T cell activity.
Abramowicz et al. [53]	2019	Human Head and Neck cancer	UM-SCC6	-	CD9, CD63, CD81	No	Yes	ZetaSizer Nano-ZS90 (Malvern)	Exosome markers upregulated by radiation; these included proteins associated with DNA repair, regulation of ROS metabolic process and de novo protein folding upregulated.
Broggi et al. [44]	2019	Melanoma	B16-F10	-	CD81, TSG 101 (CD9, CD63, ALIX, syntenin in UC plasma EVs)	No	Yes	NanoSight (Malvern)	SEC isolated fluorescently labeled EVs were demonstrated to be trafficked via lymphatics into the systemic circulation in mice. Clinical study using dUC showed a differential proteomic profile between cohorts with nodal metastasis.
Freitas et al. [55]	2019	Human gastric cancer	MKN45	-	HSP70, syntenin-1, CD9, CD63, ALIX and CD81	Cyt C	Yes	NanoSight NS300 (Malvern)	All methods were successful in isolating EVs carrying glycoproteins bearing n-linked and truncated O-linked glycans. Additionally, changing the glycosylation of the MKN45 cells impacts the glycosylation profile of isolated EVs.
Indira Chandran et al. [59]	2019	Glioma	-	Low- and high-grade Glioma (n = 82)	LC-MS showed Tetraspanins	No	Yes and immunoblot anti-SDC1	NanoSight LM10-HS (Malvern)	Plasma EV protein syndecan-1, was able as a single marker to discriminate between GBM and LGG with an AUC of 0.82 and a sensitivity and specificity of 71% and 80%, respectively.
Ludwig et al. [57]	2019	Head and Neck cancer	SCC-90, PCI-30	-	TSG101, ALIX, CD63 and CD81	No	Yes	qNano (Izon)	Differential enrichment in proteomic analysis of TEX from HPV+ and HPV- HNSCC cells identified that are biologically active e.g. CD47, CD276, MUC1.
Theodoraki et al. [47]	2019	Head and Neck Squamous cell carcinoma	-	HNSCC (n = 18), Controls (n = 5)	CD9, CD63, CD81 (flow cytometry and microarray)	No	Yes	qNano (Izon)	The TEX/total exosome ratios of disease-free patients remained low at week 14, but ratios increased (p = 0.03) in four patients who recurred. High levels of CD3−/PD-L1+ and CD3−/CTLA4+ exosomes at baseline might indicate patients who would benefit from immunotherapy
**RNA Studies**									

Rabinowits et al. [65]	2009	Lung adenocarcinoma	-	Lung Cancer (*n* = 27), Control (*n* = 9)	No	No	No	No	Using microarray but did not report candidates. Increased EV protein and RNA concentration in cancer cohort.
Sakha et al. [63]	2016	Human oral squamous cell carcinoma	HOC313-LM	-	CD9, CD63 and CD81	No	Yes	No	7 candidates (miR-17, miR-30a-3p, miR-30a-5p, miR92a, miR-181a, miR-342–3p and miR-1246) were differentially expressed oncogenic miRNAs in both cellular and exosomal data. DENND2D is a direct target of miRNA-1246 and enhanced the migration and invasion ability in co-incubated cells.
van Eijndhoven et al. [33]	2016	Classical Hodgkin lymphoma (cHL)	-	cHL (*n* = 20), Control (Healthy *n* = 9, SLE *n* = 4)	No	No	Yes	qNano (Izon)	Increased concentrations of EVs in the plasma of cHL patients compared with healthy. EV-associated miR21-5p, miR127-3p, let7a-5p, miR24-3p, and miR155-5p signals were elevated in primary and relapsed cHL patients compared with healthy individuals.
Peacock et al. [64]	2018	Oropharyngeal Squamous cell carcinoma (OPSCC)	SCC2, SCC90, SCC72, SCC90	-	CD9, CD63, TSG101	No	No	qNano (Izon)	Sequencing identified 2–15% aligned to miRNA sequences, with the rest mRNA, rRNA, tRNA, snoRNA, snRNA and lincRNA. 14 miRNA were enriched in HPV + cell-derived EVs, whereas 19 miRNAs were enriched in HPV− EVs.
				**Functional Studies**					
Wieckowski et al. [78]	2009	Head and Neck cancer, Melanoma	FasL-transduced PCI-13, Mel-SW and SLM-2	HNSCC and melanoma (*n* = 35), Control (*n* = 25),	FasL, MHC Class I, CD63, LAMP-1	No	No	No	Tumour-derived microvesicles (MVs) did not promote ex vivo proliferation of resting T-cells, impaired signalling and induced apoptosis of activated primary CD8+ T-cells and promoted proliferation of activated CD4+ T-cells.
Muller et al. [32]	2014	Not described	-	Varied	TSG101, CD81, GAPDH	No	Yes	NanoSight (Malvern)	Feasibility of miniSEC method. Morphologically intact exosomes can be successfully purified from fresh or frozen human plasma with the abilty to down-regulate CD69 expression on human activated CD4+ T cells

Schuler et al. [80]	2014	Head and Neck Squamous cell carcinoma (HNSCC)	PCI-13, Kasumi-1	HNSCC (*n* = 13), Control (*n* = 30)	CD9, CD81, CD39 and CD7	No	Yes	NanoSight (Malvern)	Plasma EVs from HNSCC patients or NC plasma carry active enzymes and can carry CD39 and CD73 proteins to Treg cells leading to immunosuppressive ADO production.
Hong et al. [69]	2016	Acute myeloid leukaemia (AML) or Head and Neck Squamous cell carcinoma	-	AML (*n* = 10), HNSCC (*n* = 5), Control (*n* = 5)	CD9, CD63 and TSG101	No	Yes	qNano (Izon)	AML exosomes co-incubated with normal human NK cells inhibit NKG2D expression levels and HNSCC exosomes suppress activation and proliferation of activated T lymphocytes.
Ludwig et al. [81]	2017	Head and Neck cancer (HNC)	-	HNC (*n* = 38) or Controls (*n* = 14)	TSG101	No	Yes	qNano (Izon)	EVs from patients with active disease induced significantly stronger apoptosis of CD8þ T cells, greater inhibition of T-cell proliferation or of NKG2D expression on NK cells and better upregulation of suppressor functions in CD4+CD39+ Treg than EVs from patients with no disease.
Ludwig et al. [82]	2018	Head and Neck Squamous cell carcinoma	PCI-13 UMSCC47, UMSCC90, SCCVII	HNSCC (*n* = 10) or Controls (*n* = 3)	TSG101	No	Yes	qNano (Izon)	Plasma-derived EVs from patients with HNSCC plasma alter HUVECs tube formation, migration, wound healing, and proliferation. Increased vascularization in murine 4-NQO orthotopic model after tumour-derived EV treatment.
Theodoraki et al. [83]	2018	Head and Neck Squamous cell carcinoma	-	HNSCC (*n* = 9), Controls (*n* = 5)	TSG 101, CD63	No	Yes	NanoSight LM-10 (Malvern)	EV cargo reflect EMT progression/regression of the parental tumour in response to PDT; and induced EMT progression/regression in recipient tumour cells following ex vivo co-incubation.
Czystowska-Kuzmicz et al. [11]	2019	Ovarian carcinoma	-	OvCa (*n* = 49), Controls (*n* = 9)	Not in SEC samples	No	Yes with Immunogold	qNano (Izon)	ARG1+ small EVs are present in ascites, as well as in the plasma of OvCa patients. Cell line-derived ARG1+ EVs impair the functions of human and murine T-cells by blocking their proliferation and reducing

									expression levels of the CD3ζ and CD3ε chains.
Sjoqvist et al. [86]	2019	Human Squamous cell carcinoma	HaCaT, CC-2509, TR146	-	CD9, Annexin 5, Flotillin	No	Yes	qNano (Izon)	Keratinocyte and fibroblast-derived exosomes altered TR146 cancer cell proliferation as well as immortalized normal skin cells HaCaT.
					**Feasibility Studies**				
Davies et al. [93]	2012	Murine Melanoma	B16BL6	-	CD9	No	Yes	No	Microfluidic platform utilising in situ photopatterned porous polymer monoliths (PPM).
Lobb et al. [41]	2015	Human squamous Non-Small-Cell Lung Cancer	SK-MES-1	Healthy (n = 1)	TSG101, CD63, Flotillin-1, HSP70, Calnexin	Albumin	Yes	qNano (Izon)	A comparison of SEC vs the kits Exo Quick and Exo Spin vs dUC. SEC was found to be optimal for clinical grade exosomes.
Welton et al. [89]	2015	Prostate cancer	Du145	Not described	TSG101, CD9, CD81 and CD63 (ELISA) CD31, MHC Class I	HSA, Apo-B	No	NanoSight LM10 (Malvern)	Comparison of Exo-Spin Midi Columns with UC. Also investigating concentration methods: CellGS Exosome precipitant with Exo-Spin kit or UC.
Suarez et al. [87]	2017	Human Melanoma	SK-MEL103	-	MHC-I (W6/32), anti-CD59 (VJ1/12), anti-CD9 (VJ1/20), anti-CD63 (TEA3/10), anti-CD81<br><br>Flow cytometry	No	Yes	NanoSight LM10 (Malvern)	Use of aldehyde-sulphate latex beads to assist the characterisation of EVs by flow cytometry
Guerreiro et al. [1]	2018	Human oral squamous cell carcinoma, Pancreatic adenocarcinoma and Human Melanoma brain metastasis	PE/CA-PJ49/E10, BxPC3, H3	-	CD9 (TSG 101, Alix, CD63, and CD81 no signal detected)	No	Yes	NanoSight NS500 (Malvern)	Use of a CELLine reactor culture flasks with SEC to isolate EVs.
Smith et al. [92]	2018	Prostate Cancer	-	n = 1 or pooled	TSG101 ELISA, Calnexin ELISA	No	CryoEM	ZetaView (Malvern)	Use of nanoscale deterministic lateral displacement (nanoDLD) arrays. Equal particle yield per input volume to qEV, better in terms of yield per time spent, or

									input volume. Also more reproducible than UC, is quicker and captures RNA expression.
Altanerova et al. [2]	2019	Various tumour cells	Primary Mesenchymal stem cells (MSCs)	-	No	No	No	NanoSight (Malvern)	Demonstration of MSC-exosomes with suicide genes represent a novel anticancer drug.
Dong et al. [92]	2019	Breast cancer	Not described	Cancer (*n* = 6), Controls (*n* = 7)	Fluorescence method used for CD63, CD81	No	Yes (SEM)	Zetaview (NTA)	Use of an integrated ExoID-Chip for efficient isolation and detection of EVs from clinical serum samples.
Lane et al. [23]	2019	Human breast cancer CCM EVs spiked into human plasma	MDA-MB-231	*n* = 1	Mass Spec	No	No	NanoSight NS300 (Malvern)	A Sepharose based SEC methodology.
Lennon et al. [3]	2019	Pancreatic ductal adenocarcinoma	PANC-1	PDAC (*n* = 5), Controls (*n* = 6)	CD63, TSG101	No	Yes	Nanosight NS300 (Malvern)	Use of quantitative single molecule localization microscopy to detect EGFR and CA19-9 content on individual EVs.
Ludwig et al. [4]	2019	Head and Neck cancer, Metastatic melanoma	UMSCC47, PCI-13, Mel526, SVEC4-10	-	TSG101, CD9, CD63, CD81 not seen in all	No	Yes	qNano (Izon)	An optimized method for the isolation of tumour-derived EVs from culture supernatants using mini-SEC method.

Abbreviations: AML - Acute Myeloid Leukaemia, BPH – Benign Prostatic Hypertrophy, CRPC – Castration Resistant Prostate Cancer, CSPC – Castration Sensitive Prostate Cancer, EM – Electron Microscopy, FasL – Fas Ligand, HNSCC - Head and Neck Squamous cell carcinoma, MHC – Major Histocompatibility Complex, NTA – Nanoparticle Tracking Analysis, OSCC - Oral Squamous cell carcinoma, OPSCC - Oropharyngeal Squamous cell carcinoma, OvCa – Ovarian Cancer, PCI-13 – Cell line, PDAC – Pancreatic Ductal Adenocarcinoma, RPM – Revolutions Per Minute, SLE – Systemic Lupus Erythematosus. Words in bold are subheadings based on broad category of study.

References:

1. Guerreiro, E.M.; Vestad, B.; Steffensen, L.A.; Steffensen, L.A.; D Aass, H.C.; Saeed, M.; Øvstebø, R.; Costea, D.E.; Galtung, H.K.; Søland, T.M. Efficient extracellular vesicle isolation by combining cell media modifications, ultrafiltration, and size-exclusion chromatography. *PLoS One* **2018**, *13*, e0204276, doi: 10.1371/journal.pone.0204276.

2. Altanerova, U.; Jakubechova, J.; Benejova, K.; Priscakova, P.; Pesta, M.; Pitule, P.; Topolcan, O.; Kausitz, J.; Zduriencikova, M.; Repiska, V., Altner, C. Prodrug suicide gene therapy for cancer targeted intracellular by mesenchymal stem cell exosomes. *Int. J. Cancer* **2019**, *144*, 897-908, doi: 10.1002/ijc.31792.
3. Lennon, K.M.; Wakefield, D.L.; Maddox, A.L.; Brehove, M.S.; Wilner, A.N. et al. Single molecule characterization of individual extracellular vesicles from pancreatic cancer. *J.Extracell. Vesicles* **2019**, *8*, doi: 10.1080/20013078.2019.1685634.
4. Ludwig, N.; Razzo, B.M.; Yerneni, S.S.; Whiteside, T.L. Optimization of cell culture conditions for exosome isolation using mini-size exclusion chromatography (mini-SEC). *Exp Cell Res* **2019**, *378*, 149-157, doi: 10.1016/j.yexcr.2019.03.014.

Publisher's Note: MDPI stays neutral with regard to jurisdictional claims in published maps and institutional affiliations.

© 2020 by the authors. Licensee MDPI, Basel, Switzerland. This article is an open access article distributed under the terms and conditions of the Creative Commons Attribution (CC BY) license (http://creativecommons.org/licenses/by/4.0/).
